# Chikungunya Virus and (Re-) Emerging Alphaviruses

**DOI:** 10.3390/v11090779

**Published:** 2019-08-24

**Authors:** Penghua Wang, Rong Zhang

**Affiliations:** 1 Department of Immunology, School of Medicine, UConn Health, Farmington, CT 06030, USA; 2 Key Laboratory of Medical Molecular Virology (MOE/NHC/CAMS), School of Basic Medical Sciences, Shanghai Medical College, Fudan University, Shanghai 200032, China

Alphaviruses belong to a family of positive sense, single-stranded RNA viruses that are transmitted mainly by mosquitoes through a blood meal and cause arthritis and/or encephalitis in humans and animals. For example, the chikungunya virus (CHIKV) causes acute and chronic crippling arthralgia and long-term neurological disorders. Since 2005, following several decades of relative silence, the CHIKV has re-emerged and caused large outbreaks in Africa, Asia, and the Americas. Since its arrival on the Caribbean Islands in late 2013, the CHIKV has caused over three million human infections, with >500 deaths directly or indirectly related to the CHIKV in Mexico, Central and South America (Data source: Pan America Health Organization). Despite the significant risk to global health posed by alphaviruses, there are no vaccines or antiviral drugs for these important pathogens. Thus, phylogenetic and epidemiological studies, clinical diagnostics, vaccine/antiviral drug development, basic research including virus-vector interaction, transmission, viral immunity, and pathogenesis are still necessary for alphaviral disease prevention. In this special issue of “Chikungunya Virus and (Re-) Emerging Alphaviruses”, we solicit 10 research articles and six review articles covering the development of vaccines and antivirals [1,2,3,4], pathogenesis/immunity [5,6,7,8], viral evolution [9], development of research/diagnostic tools [10,11], vector-virus interaction [12,13], as well as mechanisms of transmission [14]. 

Many efforts have been devoted to the development of efficacious prophylactics and therapeutics for the CHIKV, yet no specific antiviral drugs and/or licensed vaccines are currently available [15]. The review article by Jin et al. [1] have summarized recent advances in therapeutic monoclonal antibodies against the CHIKV and their mechanisms of action. López–Camacho et al. have applied a replication-deficient chimpanzee adenoviral platform, ChAdOx1, to CHIKV vaccine development. This system expresses the CHIKV structural proteins and produces CHIKV-like particles. This vaccine induces high frequencies of anti-CHIKV specific T-cell responses as well as high titers of neutralizing antibodies [3]. As alphaviruses are blood-borne and could be transmitted by blood transfusion, they pose severe safety concerns for plasma-derived medicinal products (PDMPs) in the epidemic countries. Yue et al. have described methods to inactivate/remove the CHIKV and the Mayaro virus (MAYV) from PDMPs [4]. These methods could also be a useful guide for the preparation of inactivated virus vaccines. Small molecule compounds targeting viral proteins have proven to be clinically effective at inhibiting many viruses including the human immunodeficiency virus I (HIV-1) and the hepatitis virus C (HCV), etc. Some natural herbal compounds demonstrate potent antiviral effects. Henss et al. have reported that Silvestrol, a natural compound from plants of the genus *Aglaia*, inhibits CHIKV replication and could be a potential drug candidate [2]. 

In-depth understanding of the basic mechanisms of pathogenesis and host immune responses is critical for the development of novel prophylactics and therapeutics. This is particularly true for arthritogenic alphaviral diseases, which are essentially a self-perpetuating inflammatory process initiated by viruses. Matusali et al. [15] have comprehensively reviewed the current knowledge on tissue/cellular tropism of the CHIKV, mechanisms of pathogenesis, and the spectrum of both competitive vectors and animal hosts. From a clinical angle, Armaral et al. have summarized the main hypotheses of chronic CHIK arthritis pathogenesis and therapies [5]. Barr’s review focuses on CHIKV pathogenesis in infants and children who are vulnerable to severe manifestations of CHIKV infection, such as chikungunya fever (CHIKF) and neurological sequelae. Frickmann et al. have summarized the epidemiological findings on the CHIKV in military personnel deployed in tropical settings [7]. 

Mouse models of CHIKV infection have provided many insights into CHIK arthritis pathogenesis and host immune responses. The research article by Jain et al. compares the virulence of many strains in adult C57BL/6 mice, disease pathogenesis in young (eight weeks old) and older mice (20 weeks old) [6]. The older mice are more vulnerable to CHIKV infection, accompanied by weaker immune responses. One of the CHIKV strains tested (CHIKV#01) is neurotropic and kills mice at a dose of 1X10^6^ or more viral particles. This observation is interesting because other well-studied CHIKV isolates are not lethal to immunocompetent mice. These findings highlight the potentially increasing threat of the CHIKV to humans as its virulence enhances with evolution. 

Alphaviruses, as well as many of the mosquito-transmitted flaviviruses, have existed in Africa for hundreds of years. However, only the past several decades have seen a rapid re-emergence and spread of these viruses worldwide. Global climate change, rapid urbanization, burgeoning international travel, expansion of mosquito populations, vector competence, and host and viral genetics may all contribute to the re-emergence of these viruses. Ketkar et al. have summarized the host and viral genetic determinants that could enhance viral infectivity in the host, viral fitness in mosquitoes, and viral transmission by mosquitoes [16]. A phylogenetic study by Galan–Huerta et al. describes mutations in the envelope protein that might influence virus-cell binding [9].

Advanced research tools are critical for scientific discoveries. Belarbi et al. have developed a recombinant Ross River virus (RRV) expressing a NanoLuc reporter (RRV-NLuc) for the study of physiopathological mechanisms of alphaviral arthritis [10]. This reporter virus exhibits high stability, with near native replication kinetics, which allows for real-time monitoring of viral spread and tissue tropism in vivo. Accurate diagnosis of viral infection is essential for appropriate medical treatment. Kim et al. have developed a simple and sensitive enzyme-linked immunosorbent assay (ELISA) for the serodiagnosis of CHIKV infections [11]. This assay could be applied to population-based seroprevalence survey and evaluation of vaccination efficacy in clinical trials.

Alphaviruses are transmitted to mammals mainly by mosquitoes through a blood meal [15]. It is thus important to understand the mechanisms of viral transmission and pathogenesis in vectors. Vedururu et al. have performed a whole transcriptome analysis on CHIKV-infected midguts of *Aedes albopictus* in order to identify differentially expressed genes. These results could provide preliminary clues for molecular interaction between *Aedes albopictus* and the CHIKV. Following its replication in the midgut epithelium, the CHIKV exits the midgut and infects secondary tissues including the salivary glands. Kantor et al. have investigated the pattern of CHIKV dissemination from the midgut of *Aedes aegypti* at the ultrastructural level [13]. The results suggest that the CHIKV requires a single replication cycle in the midgut epithelium before mature virions can directly traverse the midgut basal lamina during a relatively narrow time window, i.e., within 48 hrs after a blood meal. Once in the salivary glands, the CHIKV can replicate to high titers and can be then transmitted to another host during a blood meal. However, in nature the CHIKV can also pass from female mosquitoes to their offspring within the ovary or during oviposition. In a laboratory setting, Honorio et al. have demonstrated that *Ae. albopictus* mosquitoes from Brazil and Florida exhibit heterogeneous CHIKV dissemination and vertical transmission, which could contribute to outbreaks of the CHIKV and may particularly be relevant to virus survival during inter-epidemic periods [14]. 

Together, the review articles collected in this issue provide readers with a complete picture on the CHIKV diseases and research progresses. The research articles provide some novel insights into vaccine/antiviral development, research tool/diagnosis development, and also address some basic research questions such as mechanisms of viral pathogenesis, immunity, viral transmission, and evolution.
